# Water mites of the genus *Lebertia* Neuman, 1880 (Acari, Hydrachnidia, Lebertiidae) from Turkey, with the description of one new species

**DOI:** 10.3897/zookeys.238.3861

**Published:** 2012-11-05

**Authors:** Pınar Gülle, Yunus Ömer Boyacı

**Affiliations:** 1 Mehmet Akif Ersoy University, Faculty of Science and Arts, Burdur, Turkey; 2 Süleyman Demirel University, Eğirdir Fisheries Faculty, Isparta, Turkey

**Keywords:** Acari, water mites, new species, *Lebertia*, Turkey

## Abstract

A list of species of the water mitegenus *Lebertia* Neuman, 1880 known from Turkey is provided, based on bibliographical data and results from recent field work, mainly in the Southwestern part of the country. We describe one new species, *Lebertia martini* **sp. n.** and report new 28 locality records from 6 provinces for the three previously known species.

## Introduction

Water mites of the family Lebertiidae reach their maximum diversity in the Holarctic region, but isolated species have been recorded in several parts of South Asia, Africa and South America (Gerecke 2009). The genus *Lebertia* is the most species-rich member of the family. *Lebertia* species are found most frequently, and with the highest diversity, in springs and streams of temperate or boreal regions (Gerecke 2009).

Compared with intrageneric variation in other water mite genera, most *Lebertia* species are highly uniform in the shape of coxae, legs and mouth parts. Characters useful for discrimination of species and subgenera are mainly integument structures, as well as details in setation and shape of legs and palps (Di Sabatino et al. 2010).

The genus *Lebertia* is divided into 5 subgenera: *Eolebertia*, *Mixolebertia*, *Pilolebertia*, *Brentalebertia* and *Lebertia* s. str. (Gerecke 2009). So far, representatives of all subgenera except for *Brentalebertia* and *Eolebertia* have been found in Turkey. The water mite fauna of Turkey includes 240 species in 55 genera and 23 families, with 7 species of *Lebertia* known up to now (Erman et al. 2010, Bursalı et al. 2011). Here we report results of further field work, mostly done in SW Turkey, resulting in the detection of one new species (described below) and new locality records of three *Lebertia* species previously known from other parts of the country.

## Methods

Water mites were collected by hand netting and sorted on the spot from the living material, preserved in Koenike’s fluid (50% glycerin, 20% acetic acid, 30% aqua dest.) and dissected for slide mounting in Hoyer’s fluid. The composition of the material is given as males/females. All measurements are given in μm. The following abbreviations are used: Cx-I = first coxae, Cx-I mL = first coxae medial length, Dc-1-4 = dorsocentralia 1-4, H = height, L = length, n = number of specimens examined, P-1 = palp segment 1, W = width, IV-L-5 = fourth leg, fifth segment.

## Results

### Family Lebertiidae Thor, 1900. Genus *Lebertia* Neuman, 1880. Subgenus *Lebertia* Neuman, 1880

#### Lebertia (Lebertia) castalia

Viets, 1925

http://species-id.net/wiki/Lebertia_castalia

##### New records.

**Antalya Province:**21.07.2009, 37°03.03'N, 31°36.09'E, 4/4, 665m a.s.l., spring with *Fontinalis* mosses and water cress, İbradı; 09.04.2010, 36°54.84'N, 31°09.82'E, 0/2, 110m a.s.l., dense filamentous green algae, Aksu stream, Serik, collected by P. Gülle.

##### Former records from Turkey.

Muş Province (Özkan 1982); Afyon Province (Aşçı et al. 2006–2007); Erzurum Province (Boyacı and Özkan 2007).

##### Distribution.

Central, eastern and northern Europe (Gerecke 2009).

#### Lebertia (Lebertia) glabra

Thor, 1897

http://species-id.net/wiki/Lebertia_glabra

##### New records.

**Burdur Province:** 24.05.2008, 37°24.43'N, 29°48.72'E, 4/5, 1050m a.s.l., small stream feeding pond, İncekiniş, Karamanlı; 18.06.2008, 37°44.48'N, 30°31.16'E, 4/3, 980m a.s.l., stream with sandy bottom, Andık stream; 18.05.2008, 37°35.34'N, 29°54.64'E, 4/3, 1100m a.s.l., spring under agricultural and anthropogenic effect, Güneykent, Uluharman; 23.06.2008, 37°39.00'N, 30°28.48'E, 2/1, 1060m a.s.l., Aksu Spring; 17.7.2008, 37°03.47'N, 29°40.98'E, 7/2,1180m a.s.l., Kocayayla, Kozağaç; 04.08.2008, 37°45.44'N, 20°59.66'E, 2/6, 1330m a.s.l., small stream with organic pollution (small amount of farm animal feces discharge was observed), Ulupınar; 10.07.2008, 36°59.12'N, 29°29.08'E, 9/3, 1185m a.s.l., small slow flowing stream, Ballık Maşat, Altınyayla; 14.5.2008, 36°57.02'N, 29°23.01'E, 8/2, 1250m a.s.l., Elmalıyurt stream; 09.06.2008, 37°24.04'N, 30°25.05'E, 12/8, 1215m a.s.l., small spring with sandy and gravel bottom, Hasanpaşa, Tefenni; 03.06.2008, 36°59.09'N, 29°23.90'E, 5/8, 1080m a.s.l., small spring, İbecik, Altınyayla, collected by Y. Ö. Boyacı. **Isparta Province:** 16.06.2008, 37°42.56'N, 31°20.16'E, 12/20, 1290m a.s.l., small spring with gravel bottom, Pınargözü, Yenişarbademli; 13.9.2008, 38°19.15'N, 31°12.32'E, 5/8, 1060m a.s.l., spring with gravel bottom with sparse algae and aquatic plants, near trout farm, Yalvaç; 19.08.2008, 37°34.00'N, 30°52.63'E, 6/8, 945m a.s.l., fast flowing stream with clear water, Eğirdir; 22.08.2008, 37°33.23'N, 31°18.80'E, 3/6, 1300m a.s.l., spring, Yaylabeli village, Sütçüler; 21.08.2008, 37°34.00'N, 30°52.63'E, 7/14, 455m a.s.l., fast flowing stream with gravel bottom, Çandır, Sütçüler; 18.07.2008, 17.08.2008, 37°45.82'N, 31°02.00'E, 8/8, 6/8, 1190m a.s.l., fast flowing stream with gravel and sandy bottom, Köprüçay river, Pazarköy, collected by Y. Ö. Boyacı. **Antalya Province:** 18.10.2009,36°30.52'N, 32°18.16'E, 5/3, 265m a.s.l., helocrene spring, Alanya; 23.07.2009,36°43.40'N, 32°12.43'E, 4/2, 560m a.s.l., rheocrene spring with stony bottom, Alara River, Gündoğmuş, collected by P. Gülle. **Afyonkarahisar Province:** 24.05.2008, 37°51.81'N, 30°02.34'E, 9/6, 890m a.s.l., small stream with muddy bottom, Başmakçı; 18.06.2008, 38°00.14'N, 30°08.09'E, 1/3, 1125m a.s.l., Pınarlı stream, collected by Y. Ö. Boyacı. **Denizli Province:** 23.08.2008, 36°59.44'N, 29°33.62'E, 8/12, 1205m a.s.l., Gürsu stream, Çameli, collected by Y. Ö. Boyacı. **Konya Province:** 05.07.2009, 37°51.74'N, 31°38.38'E, 11/5, 965m a.s.l., stream with muddy bottom covered by algae, Üstünler, Beyşehir, Konya, collected by Y. Ö. Boyacı.

##### Former records from Turkey.

Niğde Province (as *Lebertia lineata*, Smit 1995).

##### Distribution.

West Palaearctic (Gerecke 2009).

#### Lebertia (Lebertia) maculosa

Koenike, 1902

http://species-id.net/wiki/Lebertia_maculosa

##### Former records from Turkey.

Rize Province (Pešić et al. 2007).

##### Distribution.

Central, western and southeastern Europe (Gerecke 2009).

#### Lebertia (Lebertia) schechteli

Thor, 1913

http://species-id.net/wiki/Lebertia_schechteli

##### Former records from Turkey.

Erzurum and Van provinces (as *Lebertia tuberosa*, Özkan 1982); Karaman province (Boyacı 1995); Kayseri province (as *Lebertia tuberosa*, Özkan et al. 1996); Elazığ province (as *Lebertia tuberosa*, Erman and Özkan 2000; as *Lebertia tuberosa*, Erman et al. 2006); Erzurum province (as *Lebertia tuberosa*, Boyacı and Özkan 2007).

##### Distribution.

Restricted to higher mountain ranges in western, central and southeastern Europe (Gerecke 2009). In Turkey, it is found in moderate to high altitudes.

#### Lebertia (Lebertia) martini sp. n.

urn:lsid:zoobank.org:act:EAA7D6D6-A984-4154-9FE5-74978A11971F

http://species-id.net/wiki/Lebertia_martini

##### Type series.

Holotype male, Darıbükü spring, Sütçüler, Isparta, 17.08.2008, 37°33.66'N, 31°11.84'E, 870m a.s.l., leg. Y. Ö. Boyacı. Paratypes: 4 females, same data as holotype. Paratypes: 2 female, seepage spring feeding the Köprüçay river, Pazarköy, Isparta, 22.06.2008, 37°45.82'N, 31°2.00'E, 1190m a.s.l., leg. Y. Ö. Boyacı; Paratypes: 2 female and 3 male, Gürsu spring, Çameli, Denizli, 23.08.2008, 36°59.44'N, 29°33.62'E, 1500m a.s.l., leg. Y. Ö. Boyacı. Type material dissected and slide mounted in Hoyer’s fluid, deposited at the Faculty of Fisheries, Süleyman Demirel University, Isparta, Turkey.

##### Diagnosis. 

**Figure 2.**
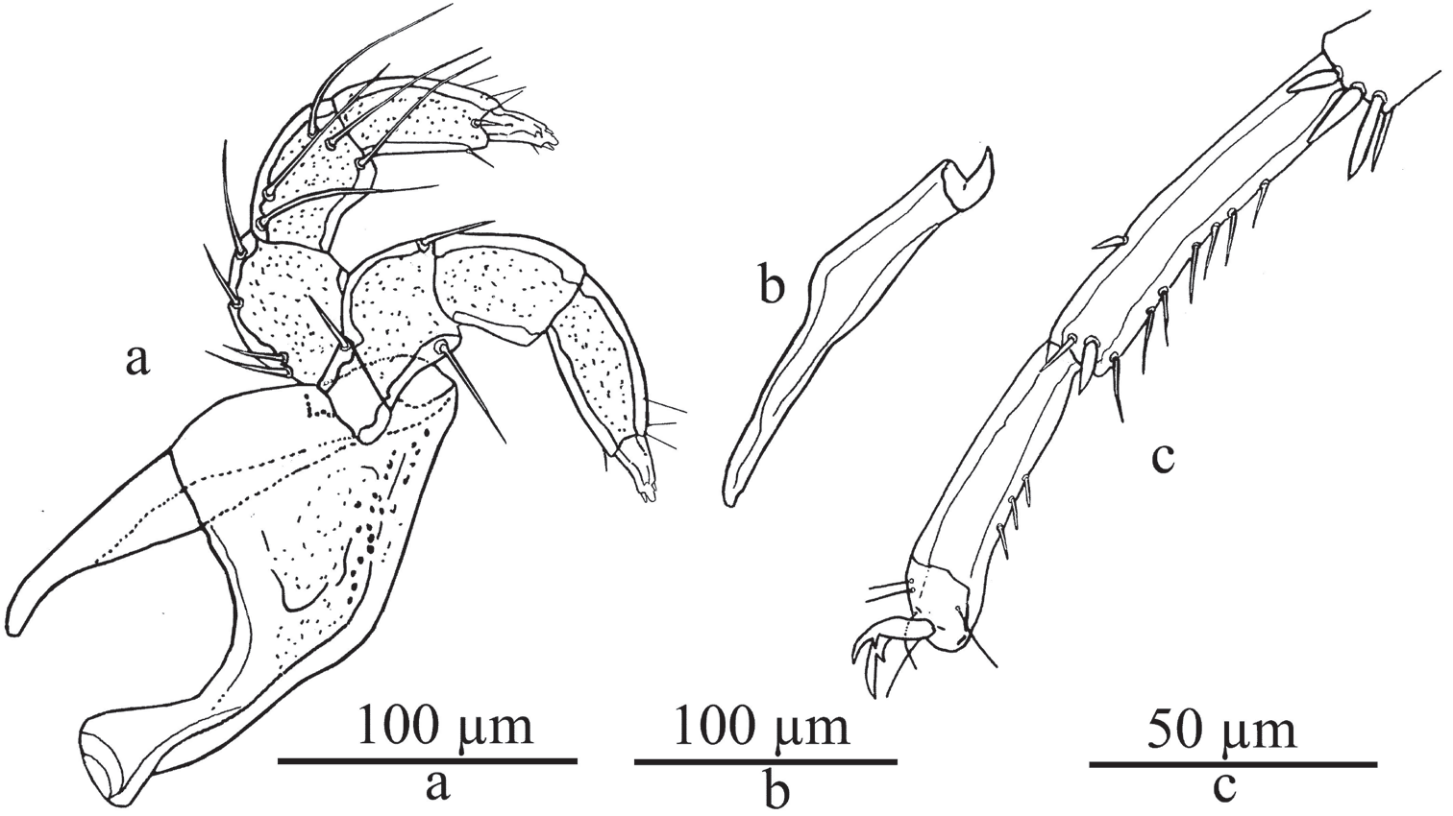
*Lebertia (Lebertia) martini* sp. n., Male: **a** gnathosoma **b** chelicera **c** IV-L-5-6.

Integument lineated. Dorsum with four paired median plates Dc-1-4 (Fig. 1a). Legs without swimming setae. Palp relatively small and stout; P-3 with paired dorsal setae located far proximally, tips of distomedial setae not extending beyond the tip of P-5, dorsodistal seta distanced from distal segment edge (Fig. 2a).

**Figure 1.** *Lebertia (Lebertia) martini* sp. n., Female: **a** idiosoma, dorsal view **b** idiosoma, ventral view, Male: **c** idiosoma, ventral view.

##### Description.

**Both sexes.** Integument dorsally and ventrally lineated. Dorsum with four paired median plates (equal in size in the both sexes) Dc-1-4, Dc-1 largest, triangular in shape and bearing the postocular setae, Dc-3 oval, Dc-2 and -4 much smaller and circular (Fig. 1a). Dorsoglandularia relatively large. Leg setation inconspicuous, no swimming setae present; number of ventral setae on IV-L-5-6: 7 and 3 respectively. Excretory pore unsclerotized. Palp relatively small and very stout; P-3 with tips of distal setae not extending beyond tip of P-5, dorsal mediodistal seta distanced from segment edge, paired dorsal setae located close together far proximally near segment base.

Male. (holotype, in parentheses variability of the paratypes given as mean, n = 3): Idiosoma L/W 275 (285)/165 (166) (Fig. 1c), integument dorsally and ventrally lineated. Capitular bay 30 (33), Cx–I mL 38 (38), capitulum 165 (171), chelicera 160 (162) (Fig. 2b), claw 14 (17) (Fig. 2c). Palp: total L 195, L/H: P-1, 17/23 (18/23); P-2, 50/47 (49/46); P-3, 53/40 (50/40); P-4, 56/25 (55/25); P-5, 19/8 (19/8). Coxae covering most of the ventral surface; posterior margin of Cx-IV smooth, not including posterior glandularia. Genital flap L 56 (58), distance between genital flap and posterior tip of the idiosoma 57 (59) (Fig. 1c). Leg segments L and total L: I-L: 22,27,23,31,39,46 = 188; II-L: 27,33,29,37,46,56 = 228; III-L: 31,37,33,44,57,55 = 257; IV-L: 40,37,45,52,68,56 = 298.

Female. (allotype,in parentheses variability of the paratypes given as mean, n = 8): Idiosoma L/W 340 (344)/200 (203). Capitular bay 33 (34), Cx-I mL 60 (61), capitulum 168 (170), chelicerae 158 (161),claw 17 (17). Palp: total L 197, L/H: P-1, 20 (21)/20 (20); P-2, 55 (55)/53 (53); P-3, 48 (48)/37 (37); P-4, 53 (54)/30 (30); P-5, 21 (22)/9 (9), distance between anterior edge of Cx-I and posterior margin of Cx-IV 307 (309). Genital flap, L 73 (74), distance between genital flap and posterior tip of the idiosoma 58 (58) (Fig. 1b). Leg segments L and total L: I-L: 23,29,25,35,42,50=204; II-L: 28,35,31,38,49,58=239; III-L: 31,37,35,49,58,57=267; IV-L: 40,38,48,55,70,71=322.

##### Discussion.

*Lebertia martini* sp. n.is the first *Lebertia* species having dorsal plates to be recorded from the Palaearctic. Presence of these plates, combined with the very stout palps will allow an easy distinction from all other *Lebertia* species in the region (Gerecke 2009). *Lebertia ventriscutata* Cook, 1974, the only further known species of the genus bearing dorsal plates was described from a spring habitat in North America (Michigan - Cook 1974), is obviously not related to *Lebertia martini*. Among others, it differs strongly in the shape of the palp and the extreme extension of the male coxal field which forms a shield completely surrounding the genital field (Cook 1974).

##### Etymology.

The species name is given in honour of the water mite specialist Dr Peter Martin (Kiel).

##### Habitat.

Crenobiontic species.

### Subgenus *Pilolebertia* Thor, 190*0*

#### Lebertia (Pilolebertia) porosa

Thor, 1900

http://species-id.net/wiki/Lebertia_porosa

##### New records.

**Burdur Province:** 20.05.2008, 37°13.17'N, 29°41.86'E, 7/10, 995m a.s.l., small stream with sandy bottom, Beyköy, Gölhisar; 20.09.2009, 37°02.16'N, 29°48.73'E, 8/13, 1400 m a.s.l., small spring with sandy bottom, Güllük, Söğüt, collected by Y. Ö. Boyacı. **Antalya Province:** 01.08.2008, 37°04.72'N, 30°34.77'E, 3/6, 305m a.s.l., reeds and reservoir channel with dense growth of submerged plants (*Ceratophyllum*), Yağca village, Kırkgöz; 29.07.2009, 37°07.37'N, 31°13.05'E, 0/2, 165m a.s.l., main stream bed, Köprüçay Beşkonak village, Manavgat, collected by P. Gülle.

##### Former records from Turkey.

Konya Province (Smit 1995); Afyon Province (Aşçı et al. 2006–2007); Erzurum Province (as *Lebertia leioderma*, Özkan 1982; as *Lebertia leioderma*, Boyacı and Özkan 2007); Elazığ Province (as *Lebertia leioderma*, Erman and Özkan 2000; as *Lebertia leioderma*, Erman et al. 2006).

##### Distribution.

Holarctic (Gerecke 2009).

#### Lebertia (Pilolebertia) insignis

Neumann 1880

http://species-id.net/wiki/Lebertia_insignis

##### Former records from Turkey. 

Tokat Province (Bursalı et al. 2011).

##### Distribution.

Central-northern Europe (Gerecke 2009).

### Subgenus *Mixolebertia* Thor, 1906

#### Lebertia (Mixolebertia) turcica

Bursalı & Özkan, 2004

http://species-id.net/wiki/Lebertia_turcica

##### Former records from Turkey.

Tokat Province (Bursalı and Özkan 2004).

##### Distribution.

Turkey (Bursalı et al. 2011).

## Conclusion

Examination of *Lebertia* material collected mainly from southwestern Turkey revealed the presence of a very distinct new species, as well as the new provincial records (28 locality from 6 province) for the three previously recorded species: *Lebertia (Lebertia) castalia* Viets, 1925 from Antalya Province; *Lebertia (Pilolebertia) porosa* Thor, 1900 from Antalya, Burdur and Konya provinces; *Lebertia (Lebertia) glabra* Thor, 1897 from Afyonkarahisar, Antalya, Burdur, Denizli, Isparta and Konya provinces. The faunistic investigation of the genus *Lebertia* in Turkey is still restricted to limited geographical regions, leaving big gaps in our knowledge of diversity of this genus in the regions of Marmara, Trakya, Eastern and Western Black Sea coast.Our results suggest that in the course of further investigations extended to cover all regions many more species will be founds.

## Supplementary Material

XML Treatment for Lebertia (Lebertia) castalia

XML Treatment for Lebertia (Lebertia) glabra

XML Treatment for Lebertia (Lebertia) maculosa

XML Treatment for Lebertia (Lebertia) schechteli

XML Treatment for Lebertia (Lebertia) martini

XML Treatment for Lebertia (Pilolebertia) porosa

XML Treatment for Lebertia (Pilolebertia) insignis

XML Treatment for Lebertia (Mixolebertia) turcica

## References

[B1] Aşçı F Bursalı A Özkan M (2006–2007) Afyonkarahisar İli Su Kenesi (Acari; Hydrachnidia) Faunası. Süleyman Demirel Üniversitesi, Eğirdir Su Ürünleri Fakültesi Dergisi 2–3 (1–2): 46–49.

[B2] Boyacı YÖ Özkan M (2007) Dumlu Çayı ve Akdağ Suyu Su Kenelerinin (Acari, Hydrachnidia) Sistematik ve Ekolojik Yönden İncelenmesi. Ege Üniversitesi Su Ürünleri Dergisi 24(1–2): 113-115

[B3] Bursalı A Özkan M (2004) A New Record of Water Mite Species *Lebertia turcica* (Lebertiidae, Hydrachnellae, Acari) from Turkey. Bulletin of Pure and Applied Sciences 23A (2): 113–116.

[B4] Bursalı A Aşçı F Özkan M (2011)*Lebertia insignis* Neuman, 1880 (Acari, Hydrachnidia, Lebertiidae), a new record for the Turkish fauna. Turkey Bulletin of Entomology 1 (1): 27– 30.

[B5] Cook DR (1974) Water Mite Genera and Subgenera. Memoirs American Entomological Institute, 21: 1-860

[B6] Di Sabatino A Gerecke R Gledhill T Smit H (2010) Hydrachnidia, Hydryphantoidea and Lebertioidea. In: Gerecke R (Ed) (2010) Chelicerata: *Acari* II. Süßwasserfauna von Mitteleuropa 7: 2-2, Elsevier GmbH, Spektrum Akademischer Verlag, München. 1−236.

[B7] Erman O Özkan M (2000) Elazığ İli Su Kenesi (Hydrachnellae, Acari) Faunası. Fırat Üniversitesi Fen ve Mühendislik Bilimleri Dergisi 12 (2): 19-28

[B8] Erman O Tellioğlu A Orhan O Çitil C Özkan M (2006) Hazar Gölü ve Behremaz Çayı Su Kenesi (Hydrachnidia: Acari) Faunası ve Mevsimsel Dağılımı. Fırat Üniversitesi Fen ve Mühendislik Bilimleri Dergisi 18 (1): 1-10

[B9] Erman O Pešić V Esen Y Özkan M (2010) A checklist of the water mites of Turkey (Acari: Hydrachnidia) with description of two new species. Zootaxa 2624: 1-48

[B10] Gerecke R (2009) Revisional studies on the European species of the water mite genus *Lebertia* Neumann, 1880 (Acari: Hydrachnidia: Lebertiidae). Abhandlungen der Senckenbergischen Gesellschaft für Naturforschung 566: 1-144

[B11] Özkan M (1982) Doğu Anadolu Su Akarları (Acari, Hydrachnellae) Üzerine Sistematik Araştırmalar-II. Atatürk Ünivertesi, Fen Fakültesi Dergisi 1: 145-163

[B12] Özkan M Erman O Boyacı YÖ (1996) Sultan Sazlığı’nın (Kayseri) Su Akarı (Hydrachnellae, Acari) Faunası Üzerine Bir Araştırma. Turkish Journal of Zoology 20: 95-98

[B13] Pešić V Ağırbaş E Turan D (2007) A contribution to the knowledge of the water mite fauna of runnig waters draining to the Eastern Black Sea coast of Turkey. Lauterbornia 59: 45-52

[B14] Smit H (1995) New records of water mites from Turkey, with 11 species new for the Turkish Fauna (Acari, Hydrachnellae). Storkia 4: 10-15

